# Juvenile idiopathic inflammatory myopathies with anti‐3‐hydroxy‐3‐methylglutaryl‐coenzyme A reductase antibodies in a Chinese cohort

**DOI:** 10.1111/cns.13658

**Published:** 2021-05-01

**Authors:** Ying Hou, Kai Shao, Bing Zhao, Tingjun Dai, Qinzhou Wang, Yuying Zhao, Chuanzhu Yan, Yaping Yan, Xiaotian Ma, Wei Li

**Affiliations:** ^1^ Research Institute of Neuromuscular and Neurodegenerative Diseases and Department of Neurology Qilu Hospital Cheeloo College of Medicine Shandong University Jinan China; ^2^ Department of Central Laboratory and Mitochondrial Medicine Laboratory Qilu Hospital (Qingdao) Cheeloo College of Medicine Shandong University Qingdao China; ^3^ Brain Science Research Institute Shandong University Jinan China; ^4^ Key Laboratory of the Ministry of Education for Medicinal Resources and Natural Pharmaceutical Chemistry National Engineering Laboratory for Resource Development of Endangered Crude Drugs in Northwest of China College of Life Sciences Shaanxi Normal University Xi'an China

**Keywords:** anti‐HMGCR antibody, juvenile idiopathic inflammatory myopathy, treatment outcome

## Abstract

**Aims:**

To characterize the clinical and histopathological characteristics and treatment outcomes of juvenile idiopathic inflammatory myopathies (JIIMs) with anti‐3‐hydroxy‐3‐methylglutaryl‐coenzyme A reductase (HMGCR) antibodies in a Chinese cohort.

**Methods:**

We detected anti‐HMGCR antibodies in a series of Chinese JIIM by ELISA and indirect immunofluorescence assay on HEK293 cells, and summarized the clinical findings of these anti‐HMGCR antibody‐positive patients.

**Results:**

Of 32 JIIM patients, 5 (15.63%) were found to be anti‐HMGCR antibody‐positive. The disease duration was 1.20 ± 0.45 months. Statin exposure was not found. Four patients had skin lesions, while typical pathological features of dermatomyositis such as perifascicular atrophy or myxovirus resistance protein A expression were not found. The mean creatine kinase level was 16771.60 U/L. Among the four patients who received long‐term (10.46 ± 1.42 years) follow‐up, three exhibited favorable outcomes with prednisone and additional immunosuppressants.

**Conclusions:**

Our study indicates that anti‐HMGCR antibodies may not be rare in Chinese JIIM. These anti‐HMGCR‐positive JIIMs were characterized by acute onset, substantially elevated creatine kinase level, and skin lesions without perifascicular changes in muscle pathology. The treatment outcome is generally favorable with the combination of steroid and immunosuppressant.

## Graphical abstract

Anti‐HMGCR antibody may not be rare in Chinese juvenile idiopathic inflammatory myopathy (JIIM). These anti‐HMGCR‐positive JIIMs were characterized by acute onset, high creatine kinase level, and skin lesions without perifascicular changes in muscle pathology. The treatment outcome is generally favorable with the combination of steroid and immunosuppressant. Anti‐HMGCR antibody testing should be seen as routine test for juvenile patients.

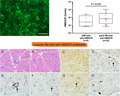

## 1 INTRODUCTION

Idiopathic inflammatory myopathies (IIMs) are a heterogeneous group of autoimmune disorders, which are characterized by proximal muscle weakness and elevated creatine kinase (CK) levels.^1^ Patients with onset age of first symptom ≤18 years are regarded as juvenile idiopathic inflammatory myopathy (JIIM).^2^

Autoantibodies recognizing 3‐hydroxy‐3‐methylglutaryl‐coenzyme A reductase (HMGCR), a target of statin medications, are typically associated with high CK levels and pathological features of necrotizing autoimmune myopathy (NAM) in adult IIM.^3^, ^4^ Clinical and pathological features of anti‐HMGCR‐positive JIIM have not yet been well characterized in the literature.^5^, ^6^, ^7^, ^8^, ^9^, ^10^, ^11^ It has been previously reported that the prevalence of anti‐HMGCR autoantibodies in JIIM varies markedly from 0.77% to 14.52%.^9^, ^10^, ^11^, ^12^ Additionally, the disease duration of anti‐HMGCR‐positive JIIM patients could also vary substantially. For instance, in two series studies with Japanese cohorts, one showed that more than half of the patients with anti‐HMGCR antibody had disease duration of more than 2 years, and the other study showed that all 5 anti‐HMGCR‐positive JIIM had over 3 years of disease duration and 4 of these 5 patients even had disease course longer than 10 years of mimicking muscular dystrophy (MD),^8^, ^11^ while two separate studies of Chinese and American cohorts reported 60% and 100% anti‐HMGCR‐positive JIIM showed acute onset, respectively.^5^, ^10^ Consistently, most previous research showed extremely high serum CK level (≥ 8000 U/L) in anti‐HMGCR‐positive JIIM patients, with a record of over 20000 U/L in a study with European patients.^5^, ^7^, ^9^, ^11^, ^13^, ^14^, ^15^, ^16^, ^17^ However, it is noted that a Japanese study reported that the CK level of anti‐HMGCR‐positive JIIM was merely about 2000 U/L.^8^ While multi‐immunotherapy has been reported to be a common treatment method for anti‐HMGCR‐positive JIIM,^9^, ^10^ a few recent cases showed that intravenous immunoglobulin (IVIG) could also lead to dramatic clinical responses. In some cases, no medication was required.^14^, ^15^, ^17^ Indeed, these inconsistent reports in the literature have led to confusion for devising appropriate treatments for this condition, calling for more clinical research.

In this study, we explored the prevalence of anti‐HMGCR autoantibodies among 32 JIIM patients and summarized the clinical, serological, and histopathological characteristics and treatment outcomes of 5 anti‐HMGCR‐positive patients to improve the diagnosis and treatment of this disease.

## 2 PATIENTS AND METHODS

### 2.1 Participants

Clinical data were collected from 227 patients with IIM in the Department of Neurology at Qilu Hospital of Shandong University from April 2005 to September 2019. The diagnosis and classification of IIM were based on the criteria proposed by the European Neuromuscular Centre.^18^ Among them, 32 were defined as JIIM patients whose onset age of first symptom was ≤18 years.^2^ All the patients were Chinese in origin and assessed by two IIM experts. We reviewed their clinical manifestations, laboratory findings, muscle pathological features, treatment regimens, and outcomes. In order to establish an optimized in‐house cutoff for anti‐HMGCR antibody detection and to compare the clinical sample, we also studied a healthy control (HC) group of 100 age‐ and sex‐matched individuals who received a routine health examination and for whom other diseases were excluded. Serum samples were collected from all IIM patients and HCs, and stored at −80°C until measurements.

In regard to clinical assessments, “chronic disease duration” was defined as a duration over 12 months from the disease onset to first examination.^19^ Muscle strength was evaluated by the ordinal six‐point (0–5) manual muscle testing (MMT) scale; asymmetric muscle weakness was defined as no less than 1 grade measured by MMT between two sides of the same muscle group or the same joint activity.^20^ Treatment outcomes were graded as follows: no improvement, mild improvement (1 grade improvement in at least one muscle group, persistently requiring assistance in daily activities), moderate improvement (>1 grade in multiple muscle groups, occasionally requiring assistance in daily activities), marked improvement (only mild weakness without functional impairment), or return to baseline (no symptoms or signs of muscle weakness); a favorable outcome was defined as marked improvement or return to baseline.^21^

The study was performed in accordance with the Declaration of Helsinki. All patients or their guardian gave their written informed consent, which was approved by the Ethics Committee of Qilu Hospital (Qingdao), Shandong University, China (KYLL‐qdql2020019).

### 2.2 Myositis‐specific antibody (MSA) detection

Anti‐HMCGR antibodies were measured in 227 patients with IIM and 100 HCs with QUANTA Lite^®^ HMGCR Kit (Inova Diagnostics), following the manufacturer's instructions.^22^ We defined and established the in‐house cutoff value at the 99th percentile of values in the HC group.

All the positive samples from IIM patients and 40 age‐ and sex‐matched negative samples (20 IIM patients and 20 HCs) were retested by indirect immunofluorescence assay (IIFA) on HEK293 cells to verify the accuracy of results. The detection of anti‐HMGCR antibodies by IIFA was performed following the protocols described in previous reports ^23^, ^24^ with minor modifications. Specifically, 36 hours after the transfection with a plasmid carrying human HMGCR gene, HEK293 cells were fixed with 4% paraformaldehyde and permeabilized with 0.4% Triton X‐100 in phosphate‐buffered saline (PBS) for 10 mins. The fixed cells were incubated with serum samples, which were diluted with PBS at the ratio of 1:10, for 30 mins at room temperature. After washed with PBS, the cells were incubated with fluorescein isothiocyanate–labeled goat anti‐human IgG antibody (Jackson ImmunoResearch Inc.) for 30 mins at room temperature. Immunostained cells were screened with fluorescence microscope (EVOS M5000; Thermo Fisher Scientific Inc.).

All 32 patients with JIIM were also tested by line immunoassay (Euroline Myositis Profile 3 ImmunoLINE‐Blot; Euroimmun) for other MSAs including anti‐signal recognition particle (SRP), anti‐EJ, anti‐Jo‐1, anti‐OJ, anti‐PL‐12, anti‐PL‐7, anti‐Mi‐2, anti‐melanoma differentiation‐associated gene 5 (MDA 5), anti‐transcriptional intermediary factor 1γ (TIF1γ), anti‐nuclear matrix protein 2 (NXP 2), and anti‐small ubiquitin‐like modifier‐activating enzyme (SAE).

### 2.3 Muscle biopsy

Muscle biopsies were taken from the biceps brachii, the deltoid, or the quadriceps of all 32 patients with JIIM. Serial frozen sections were stained with hematoxylin and eosin (HE), anti‐CD3 mouse monoclonal antibody (clone LN10; Zhongshan Golden Bridge Biotechnology), anti‐CD8 rabbit monoclonal antibody (clone SP16; Zhongshan Golden Bridge Biotechnology), anti‐major histocompatibility complex class I (MHC‐I) rabbit monoclonal antibody (clone EP1395Y; Abcam), anti‐major histocompatibility complex class II (MHC‐II) mouse monoclonal antibody (clone CR3/43; Dako), anti‐membrane attack complex (MAC) mouse monoclonal antibody (clone aE11; Dako), and anti‐myxovirus resistance protein A (MxA) rabbit polyclonal antibody (Abcam). The muscle CD3+ T lymphocytic infiltrates were classified as scattered or focal: scattered infiltrates were defined as more than 15 CD3+ T cells scattered in the endomysium, perivascular, and/or perimysial region per high‐power field (objective 20×) ^25^ ; focal infiltrates referred to more than 15 clustered CD3+ T cells (objective 20×), which were also identifiable on HE staining sections. The sarcoplasmic MxA expression was defined as the cytoplasmic positivity of MxA in non‐necrotic fibers.

### 2.4 Statistical analysis

Qualitative variables were expressed as percentages and absolute frequencies, while quantitative features were reported as mean and standard deviation (SD) values. The Shapiro‐Wilk normality test was used for checking the assumption of normal distribution. The Mann‐Whitney U test or Student's *t* test was applied to continuous data. Categorical variables were analyzed by Fisher's exact test as predicted frequency <5. Statistical significance was defined as *p* < 0.05. All analyses were performed using SPSS 22.0 (IBM Corp.).

## 3 RESULTS

### 3.1 Detection of anti‐HMGCR antibody in JIIM patient

**FIGURE 1:**
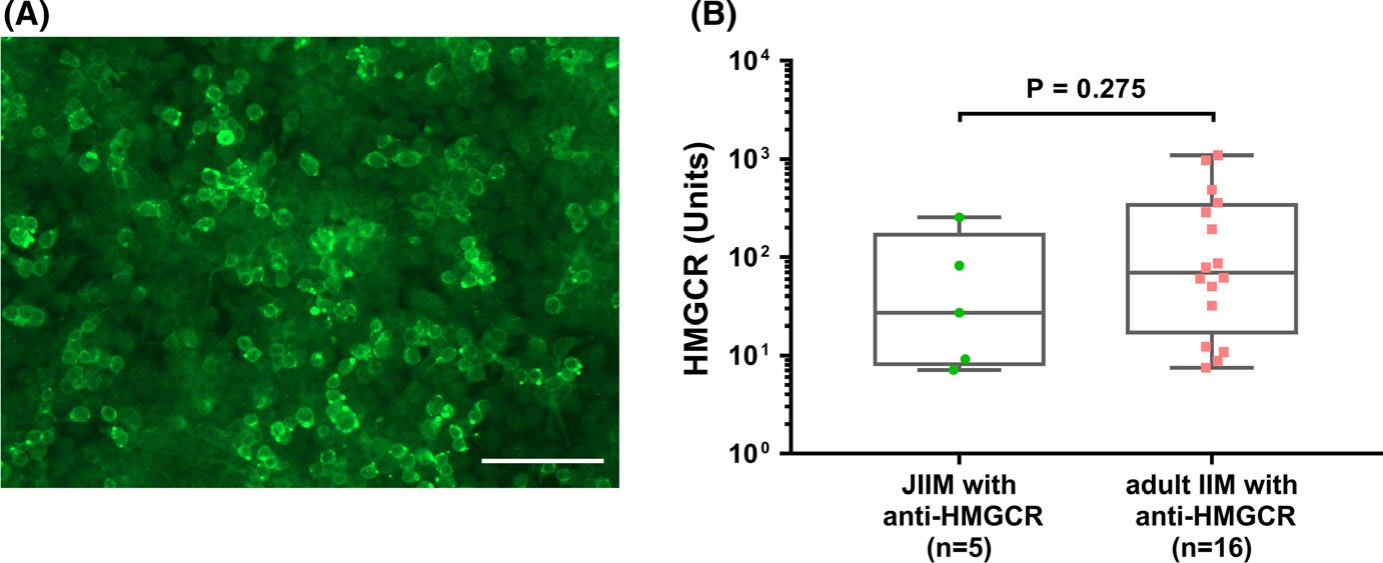
Detection of anti‐HMGCR antibodies. (A) IIFA pattern of anti‐HMGCR antibody on HEK293 cells showed a membrane fluorescence staining (scale bar = 100 μm). (B) Anti‐HMGCR antibody titers by ELISA in different groups: JIIM with anti‐HMGCR and adult IIM with anti‐HMGCR

Anti‐HMGCR antibodies were detected in 5 (15.63%) of 32 patients with JIIM, and all these 5 patients were anti‐HMGCR‐positive by confirmatory IIFA (Figure 1A). As anti‐HMGCR antibodies were found in 16 adult IIM patients, the frequency of anti‐HMGCR antibodies did not show significant difference between adult IIM and JIIM patients (*p* = 0.189). Moreover, the titer of anti‐HMGCR antibody also did not show significant difference between these two groups (*p* = 0.275) (Figure 1B).

### 3.2 Clinical features in anti‐HMGCR‐positive JIIM patients

The clinical features of the 5 JIIM patients with anti‐HMGCR antibody are summarized in Table 1. All these 5 patients were female with age at onset ranging from 4 to 15 years (mean ± SD, 10.20 ± 4.55 years). The duration from disease onset to the first visit was all within 2 months (mean ± SD, 1.20 ± 0.45 months). Notably, none of these 5 patients had prior exposure to statin or statin‐containing foods (including oyster, mushrooms, red yeast rice, or pu‐erh tea). Skin lesions were seen in four patients. Specifically, two patients had Gottron's sign, which was the typical rash of DM, 1 with hypopigmentation in the forearms and one with malar rash. Besides, all five patients experienced proximal limb weakness, two patients with neck weakness, and one patient with asymmetric muscle weakness. Dysphagia was seen in one patient and myalgia in two patients. Except for skin lesions, no extramuscular manifestations such as malignancy, interstitial lung disease (ILD), cardiac involvement, rheumatic disease, or Raynaud's phenomenon were found in these five patients.

**TABLE 1:** Clinical and histopathological features of 5 anti‐HMGCR‐positive JIIM patients

Patient	Sex/age(y)/disease duration (mo)	Statin use	Muscle symptoms	Extramuscular symptoms	MMT in the weakest muscle	Highest CK (U/L)	Inflammation	PA/PN	MxA expression	MHC‐I/MHC‐II	MAC capillary/sarcolemmal
1	F/12/1	–	PW, DW	Gottron's sign	3	7191	Scattered, focal	−/−	–	Focal/–	+/−
2	F/13/2	–	PW, NW, AW, DW	Gottron's sign	3	4231	Scattered	−/−	–	Focal/–	−/+
3	F/4/1	–	PW,	Hypopigmentation	2	13470	Focal	−/−	NS	Diffuse/‐	−/−
4	F/15/1	–	PW, NW, dysphagia, myalgia, DW	–	3	38966	–	−/−	–	Focal/–	−/+
5	F/7/1	–	PW, myalgia	Malar rash, erythema	3	20000	Focal	−/−	–	Diffuse/–	+/+

Abbreviations: AW, asymmetric muscle weakness; CK, creatine kinase; DW, distal weakness; F, female; HMGCR, 3‐hydroxy‐3‐methylglutaryl‐coenzyme A reductase; MAC, membrane attack complex; MHC‐I, major histocompatibility complex class I; MHC‐II, major histocompatibility complex class II; MMT, manual muscle testing; MxA, myxovirus resistance protein A; NS, not stained; NW, neck weakness; PA, perifascicular atrophy; PN, perifascicular necrosis; PW, proximal weakness.

The CK level in serum was markedly elevated in all five JIIM patients with anti‐HMGCR antibody (mean ± SD, 16771.60 ± 13810.95 U/L). No other MSAs were found in these patients.

### 3.3 Histopathological findings of muscle biopsies

**FIGURE 2:**
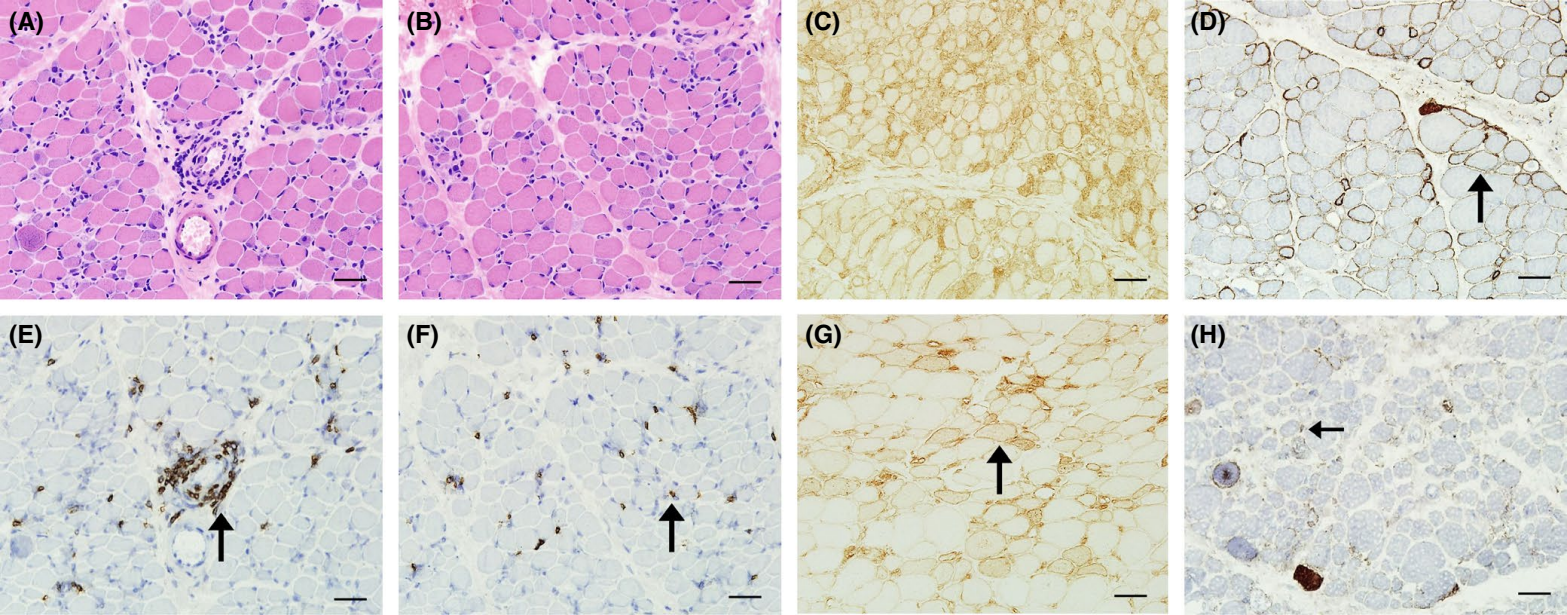
Muscle pathology of anti‐HMGCR‐positive JIIMs. (A, E) Focal lymphocytic infiltrates in Patient 1 (black arrow). (B, F) Scattered lymphocytic infiltrates in JIIM patients with anti‐HMGCR antibody in Patient 1 (black arrow). (C) Diffuse MHC‐I expression in Patient 3. (G) Focal MHC‐I expression in Patient 4 (black arrow). (D) MAC deposition in sarcolemma of non‐necrotic fiber in Patient 4 (black arrow). (H) MAC deposition in endomysial capillaries in Patient 5 (black arrow). Scale bars = 50 μm

On muscle pathology, all five patients showed mild endomysial fibrosis, necrosis, and regeneration of muscle fibers with widespread variation in fiber size. The muscle lymphocytic infiltrates were seen in 80.00% (4/5) of anti‐HMGCR‐positive JIIM patients. The infiltrates were scattered in one patient, focal in 2 patients, and both scattered and focal in one patient. All these 4 patients with CD3+ T‐cell infiltration also showed CD8+ T‐cell expression, and there was a combination of CD8+ T cells and CD4+ T cells both in focal and in scattered. Neither the perifascicular atrophy, perifascicular necrosis, sarcoplasmic MxA expression, MHC‐II expression nor perifascicular MHC‐I expression was seen. Obvious MAC deposition in sarcolemma of non‐necrotic muscle fibers was found in 3 patients. Two patients showed MAC‐positive staining on a few endomysial capillaries, although the density was dramatically less than the typical DM. MHC‐I was diffusely expressed in 2 patients and focally expressed in non‐necrotic fibers in 3 patients (none with perifascicular distribution) (Figure 2).

### 3.4 Treatment and outcomes

In the present study, among 5 anti‐HMGCR‐positive JIIM, 4 patients were followed up for more than 3 years (mean ±SD, 10.46±1.42 years). The detailed drug therapy and treatment outcomes of these 5 anti‐HMGCR‐positive patients are summarized in Table 2. One patient with marked improvement was not followed up after 30 months of treatment with oral prednisone. With the 4 patients who undergo a long‐time follow‐up, they were all initially treated with oral prednisone (1 mg/kg/day) and received additional immunotherapies including IVIG, cyclophosphamide, methotrexate, and azathioprine subsequently. All 4 patients suffered from relapse during taper or discontinuation of the drugs. While only one patient was drug‐free at the last follow‐up, they all showed improvement of muscle strength and decrease in CK level. In addition, 3 (75.00%) of these 4 patients had favorable outcomes.

**TABLE 2:** Drug therapy and outcomes of 5 anti‐HMGCR‐positive JIIM patients

Patient	Clinical course (y)	Drugs	Relapse	Drugs at last follow‐up	MMT at last follow‐up	CK at last follow‐up (U/L)	Outcomes
1^a^	2.50	Pred	No	Pred 5 mg/qd	4	210	Marked improvement
2	10.53	Pred IVIG MTX	Yes	Pred 7.5 mg/qd	3	230	Mild
3	9.77	Pred IVIG MTX	Yes	Pred 7.5 mg/qd MTX 7.5 mg/qw	4	500	Marked improvement
4	12.41	Pred IVIG MTX AZA CTX	Yes	Medicine‐free	5	68	Return to baseline
5	9.13	Pred IVIG MTX	Yes	Pred 5 mg/qod MTX 7.5 mg/qw	4	330	Marked improvement

Abbreviations: AZA, azathioprine; CK, creatine kinase; CTX, cyclophosphamide; HMGCR, 3‐hydroxy‐3‐methylglutaryl‐coenzyme A reductase; IVIG, intravenous immunoglobulin; JIIM, idiopathic inflammatory myopathy; MMT, manual muscle testing; MTX, methotrexate; Pred, prednisone.

^a^ Patient 1 was not followed up after 2.5 years of treatment.

## 4 DISCUSSION

This is the first study to evaluate the prevalence of anti‐HMGCR antibody in a large Chinese cohort of JIIM patients. This study comprehensively analyzed the clinical features and treatment outcomes of these anti‐HMGCR‐positive patients. The study indicated that anti‐HMGCR antibodies are not rare in Chinese JIIM patients. The clinical characteristics include acute disease duration, extremely high CK level, without extramuscular manifestations except for skin lesion. Skin involvement was usually found despite the rare occurrence of typical DM pathological features such as perifascicular atrophy, sarcoplasmic MxA expression, or obvious MAC‐positive capillary. Although anti‐HMGCR‐positive JIIM patients always needed additional immunotherapy besides prednisone, most of them were able to receive favorable outcomes. Based on these findings, we recommend making the test of anti‐HMGCR antibody a routine test for juvenile patients with acute disease onset and very high CK level.

In our study, we identified 5 JIIM patients with anti‐HMGCR antibodies. The prevalence of anti‐HMGCR antibodies in our JIIM cohort is 15.63%, which is similar to that in Japan (14.52%),^11^ but substantially higher than that in America (1.14%) and the United Kingdom (UK) (0.77%‐1.05%).^9^, ^10^, ^12^ The observed low prevalence statistics in America and the UK may be attributable to Bohan and Peter inclusion criteria of low specificity. Nevertheless, there is marked variability across ethnic groups in terms of the prevalence of anti‐HMGCR antibodies. While anti‐HMGCR antibody is closely related to statin exposure in Western countries, it is noted that statin exposure did not occur in anti‐HMGCR‐positive JIIM patients expect for one Japanese patient.^6^, ^7^, ^8^, ^26^, ^27^, ^28^ Consistently in the present study, the included anti‐HMGCR‐positive JIIM patients had no prior exposure to statin or statin‐containing foods including oyster mushrooms, red yeast rice, or pu‐erh tea, indicating the presence of alternative disease triggers in JIIM cohort.

In our JIIM cohort, the clinical features of anti‐HMGCR‐antibody‐positive patients were distinct. Firstly, they were predominantly females, which is similar to a previous Chinese study.^5^ Importantly, unlike many reported cases with chronic progressive disease onset mimicking MD,^8^, ^11^, ^14^, ^17^ patients in our cohort generally showed acute disease duration less than 2 months, indicating that anti‐HMGCR‐positive JIIM should be considered in pediatric patients with acute disease duration. In addition, the prevalence of skin rashes in Chinese JIIM patients with anti‐HMGCR antibodies was more than 60%, which was in line with the statistics reported in America (about 80%) but considerably higher than that reported in Japan (about 20%).^5^, ^8^, ^10^, ^11^ Furthermore, our study suggested that non‐typical DM skin rashes can be common in anti‐HMGCR‐positive JIIM patients, and careful assessments of skin condition should be performed. With regard to the laboratory examination of JIIM patients with anti‐HMGCR antibody, the serum CK level was extremely high, consistent with previous studies.^5^, ^7^, ^9^, ^11^, ^17^ Moreover, our patients with anti‐HMGCR antibody did not show positivity for any other MSAs, indicating that MSAs are generally mutual exclusive.

The histopathological features of anti‐HMGCR‐positive JIIM also appear to be distinctive. Although most anti‐HMGCR‐positive patients in the study showed DM/DM‐like skin lesions, the histological pattern was not classic for typical DM pathological features as none of perifascicular atrophy, MxA‐positive fibers, perifascicular expression of MHC‐I, or obvious MAC‐positive capillary was detected. Besides, unlike anti‐HMGCR‐positive adult IIM patients showed few inflammation infiltration,^3^, ^4^ the JIIM patients with anti‐HMGCR antibody had high frequency of lymphocytic infiltrates. CD8+ T cells expressed less than CD3+ T cells in all four patients with lymphocytic infiltration, and there was a combination of CD8+ T cells and CD4+ T cells in these four patients. Thus, anti‐HMGCR‐positive JIIM should be considered as a new distinct subtype. Indeed, unlike many reported cases with marked endomysial fibrosis,^11^, ^14^ only mild endomysial fibrosis was found in our patients, which may attribute to the acute disease onset and the early performance of muscle biopsy.

In our study, JIIM patients with anti‐HMGCR antibodies generally had acute disease onset, skin lesions, and extremely high CK level without typical dermatomyositis (DM) pathological features. These clinical and histopathological manifestations indicate that anti‐HMGCR‐positive JIIM should be regarded as a distinct subgroup of JIIM in Chinese cohort.

Additionally, we found that our patients had good prognosis, though most required prednisone and additional immunotherapies. One explanation for the favorable outcome may be the acute disease onset as improvements were notable in patients with shorter duration.^17^ Besides, weakness relapse was commonly found in JIIM patient with anti‐HMGCR antibody since younger patients were more likely to have refractory disease in anti‐HMGCR myopathy.^27^ Moreover, immunosuppressant should be given timely in anti‐HMGCR‐positive JIIM to achieve favorable prognosis since JIIM with anti‐HMGCR antibody generally need multi‐immunotherapy.

A limitation of our present study is that anti‐HMGCR antibody was not tested by the gold standard immunoprecipitation.^7^, ^26^ However, the high concordance between the commercial ELISA and IIFA on HEK293 cells should assure our results. Another limitation relates to the small sample size of JIIM patients with anti‐HMGCR antibodies. Future studies, in particular comparative studies, are required to confirm clinical features and treatment outcomes of anti‐HMGCR‐positive patients.

## 5 CONCLUSIONS

Anti‐HMGCR antibodies may not be rare in Chinese JIIM patients. These anti‐HMGCR‐positive patients typically showed acute disease onset, skin lesions, and extremely high CK level without typical DM pathological feature. The treatment of multi‐immunotherapy generally leads to favorable outcomes. We suggest that anti‐HMGCR antibody should be routinely tested in juvenile patients with acute disease onset and extremely high CK level, as an approach to assist timely diagnosis and treatment. Given that our present study is a retrospective review of a relatively small sample of anti‐HMGCR‐positive JIIM patients, future studies based on larger cohorts of patients are required to support our conclusions.

## CONFLICTS OF INTEREST

None.

## AUTHOR CONTRIBUTIONS

YH and KS contributed equally to this work. YH contributed to study design, evaluation of the patients, data acquisition, interpretation and analysis, and manuscript drafting. KS contributed to MSA testing and manuscript revising. BZ, TD, QW, YZ, CY, YY, and XM contributed to data acquisition. WL contributed to study design, result interpretation, and manuscript revising. All authors approved the version of the article to be published.

## Data Availability

All data relevant to the study are either included in the article or will be shared at the request of any qualified investigator.
